# White matter correlates of cognitive domains in normal aging with diffusion tensor imaging

**DOI:** 10.3389/fnins.2013.00032

**Published:** 2013-03-13

**Authors:** Efrat Sasson, Glen M. Doniger, Ofer Pasternak, Ricardo Tarrasch, Yaniv Assaf

**Affiliations:** ^1^Department of Neurobiology, Faculty of Life Sciences, Tel Aviv UniversityTel Aviv, Israel; ^2^Department of Clinical Science, NeuroTrax CorporationBellaire, TX, USA; ^3^Psychiatry Neuroimaging Laboratory, Department of Psychiatry, Brigham and Women's Hospital, Harvard Medical SchoolBoston, MA, USA; ^4^School of Education, Tel Aviv UniversityTel Aviv, Israel

**Keywords:** magnetic resonance imaging, diffusion tensor imaging, executive function, information processing speed, memory, aging, white matter, temporal lobe

## Abstract

The ability to perform complex as well as simple cognitive tasks engages a network of brain regions that is mediated by the white matter fiber bundles connecting them. Different cognitive tasks employ distinctive white matter fiber bundles. The temporal lobe and its projections subserve a variety of key functions known to deteriorate during aging. In a cohort of 52 healthy subjects (ages 25–82 years), we performed voxel-wise regression analysis correlating performance in higher-order cognitive domains (executive function, information processing speed, and memory) with white matter integrity, as measured by diffusion tensor imaging (DTI) fiber tracking in the temporal lobe projections [uncinate fasciculus (UF), fornix, cingulum, inferior longitudinal fasciculus (ILF), and superior longitudinal fasciculus (SLF)]. The fiber tracts were spatially registered and statistical parametric maps were produced to spatially localize the significant correlations. Results showed that performance in the executive function domain is correlated with DTI parameters in the left SLF and right UF; performance in the information processing speed domain is correlated with fractional anisotropy (FA) in the left cingulum, left fornix, right and left ILF and SLF; and the memory domain shows significant correlations with DTI parameters in the right fornix, right cingulum, left ILF, left SLF and right UF. These findings suggest that DTI tractography enables anatomical definition of region of interest (ROI) for correlation of behavioral parameters with diffusion indices, and functionality can be correlated with white matter integrity.

The ability to perform complex as well as simple cognitive tasks engages a network of brain regions mediated by the white matter fiber bundles connecting them. Different cognitive tasks employ distinctive white matter fiber bundles. For example, memory involves the temporal lobe white matter projections connecting the hippocampus with other brain structures.

Aging is accompanied by deterioration of cognitive functions, as well as changes in gray and white matter integrity. Cross sectional and longitudinal behavioral studies have found significant declines in various abilities, including encoding and retrieval of new memories, working memory, executive functions, processing speed and spatial ability (Craik et al., 1987; Petersen et al., 1992; Youngjohn and Crook, 1993; Small, 2001). Several imaging studies tried to relate diffusion parameters measured in white matter fiber systems and age related cognitive decline. It was shown that reduced integrity of specific fiber tracts are differentially correlated with declines in components of executive functions, including working memory, problem-solving, categorical task switching and Stroop color-word interference (Sullivan et al., 2006; Madden et al., 2009; Zahr et al., 2009). In cortical association fiber tracts, correlation was found with decrement in set-shifting performance observed with age (Perry et al., 2009). Motor scores were correlated with FA or diffusivity in several lateral fiber bundles and in several callosal sectors (Sullivan et al., 2010). DTI parameters in the ILF and IFOF were correlated differently with scores in different visual tasks, and posterior projections of the corpus callosum (CC) were found to correlate to memory and executive functions (Voineskos et al., 2012). Voxel-wise correlation yielded a significant negative association between FA in the body of the CC and performance in a complicated motor task (Koch et al., 2012).

Most of the above mentioned DTI studies used averaged DTI parameters along the fiber tracts. However, DTI parameters can be heterogeneous along the tract and the spatial information obtained from the fiber tracking procedure may be important for more accurate understanding of the relationship between cognitive function and white matter integrity. Deterioration of brain tissue during aging involves the temporal lobe, along with the cognitive functions it subserves. The temporal lobe has several white matter projections: the cingulum is known to be involved in many brain functions, such as working memory (Sepulcre et al., 2009), and attention (Nestor et al., 2007; Hamilton et al., 2008); The fornix is important for learning and formation of new memories (Spiers et al., 2001); The superior longitudinal fasciculus (SLF) has an important role in higher brain functions, more particularly language and language disorders (Lichtheim, 1885; Damasio and Damasio, 1980; Tanabe et al., 1987; Catani et al., 2003; Wise, 2003; Geldmacher et al., 2007); the inferior longitudinal fasciculus (ILF) is known to play an important role in visual memory as demonstrated by post mortem studies (Bauer and Trobe, 1984), lesion studies (Shinoura et al., 2007) and imaging studies in congenital prosopagnosia (Thomas et al., 2009) and face recognition (Tavor et al., 2010); the uncinate fasciculus (UF) plays an important role in the formation and retrieval of memories as shown by lesion (Levine et al., 1998) and animal studies (Squire et al., 2004).

In the present paper we used the inter-subject variability in various cognitive domains to relate cognitive performance and white matter integrity in five temporal projections: the UF, fornix, cingulum, ILF, and SLF. We computed voxel-wise correlations between cognitive performance in higher-order cognitive domains (executive function, information processing speed, and memory) and white matter integrity, as measured by ADC, and FA along the different temporal fiber tracts, correcting for age and motor ability.

## Methods

### Subjects

Subjects were 52 healthy volunteers, age 25–82 years, all right handed, 20 males and 32 females. Mean/median age was 51 years. Age distribution was approximately uniform across the age range. The local Tel Aviv-Sourasky Medical Center Helsinki Committee approved the research protocol, and all participants signed an informed consent. Subjects had no history of neurological diseases or psychological disorders, did not use neuropsychiatric medication, had no history of drug or alcohol abuse, and had intact vision. All subjects were right handed and had at least 15 years of education. Volunteers with common age-related diseases such as diabetes, high blood pressure, and high levels of cholesterol were not excluded from the cohort.

### Neuropsychological tests

Participants underwent a series of computerized cognitive tests (Mindstreams, NeuroTrax Corp., Bellaire, TX) that evaluate performance across an array of cognitive domains known to deteriorate during aging (memory, executive function, visual spatial processing, verbal function, attention, information processing speed, and motor skills) and provide measurements of accuracy and reaction time (RT) shown to be valid and reliable (Russell, 2011). These tests have been used in clinical settings, as well as in studies of normal aging relating these neuropsychological measurements to, e.g., genetic findings (Thaler et al., 2012), psychiatric and neurodegenerative disorders (Doniger et al., 2006; Plotnik et al., 2011), and imaging parameters (Sasson et al., 2010, 2012). In the present study, we performed factor analysis of 44 cognitive outcome parameters. A detailed description of the cognitive tests used in the correlation analysis with DTI parameters appears below (in brief). A more detailed description of the cognitive tests was published previously (Sasson et al., 2012).

### Factor analysis procedure

Factor analysis was performed using SPSS software (SPSS Inc., Chicago, IL, USA), as follows:

1. Factor analysis of 44 cognitive outcome parameter scores using principal component analysis (PCA) with orthogonal (VARIMAX) rotation and limiting the number of factors to 5 (although 10 factors obtained eigenvalues higher than 1) that explained 65% of the items total variance. The number of factors selected corresponded to the point of inflexion in the respective scree plot. The percentage of variance explained derives from that point.
2. Based on the rotated component matrix, and as we aimed to extract “pure” factors, we dismissed items with a difference below 0.1 between the 2 highest loadings. 14 scores were dismissed.
3. Each factor contained cognitive scores with loadings greater than 0.4 as accepted in the PCA literature (Stevens, 2002).
4. Cronbach's alpha was used to assess the internal reliability of the factors. Factors with a Cronbach's alpha of <0.8 were excluded, resulting in the exclusion of two factors and the retention of three.
5. The three factors retained were comprised of 18 cognitive scores. The scores and their factor loadings are shown in Table 1. Factor loadings that correspond to the specific domain are shown in bold. Each factor was assigned a name describing its constituent scores: executive function, memory, and information processing speed.
6. Calculation of factor scores: for each factor, the *z*-scores of the tests comprising it were averaged to give the factor score. These factor scores served as a covariate input for the DTI indices.

**Table 1:** **Factor analysis weights of final 20 cognitive measures, assigned to each cognitive domain**.

	**Executive function**	**Memory**	**Information processing speed**
Stroop WI RT	**0.82**	−0.15	0.32
Stroop WI RD	**0.80**	−0.14	−0.22
GoNoGo SD	**0.80**	−0.17	0.12
Stroop NOI1 RT	**0.74**	−0.33	0.10
GoNoGo RT	**0.72**	−0.11	0.33
Stroop NOI1 SD	**0.62**	0.07	0.13
NV Memory im rep3	−0.20	**0.87**	−0.14
NV Memory im rep4	−0.27	**0.86**	−0.18
NV Memory Delayed	−0.28	**0.85**	−0.14
NV Memory im rep2	−0.21	**0.82**	−0.17
NV Memory im rep1	0.037	**0.71**	−0.16
Verbal Memory im rep 1	−0.11	**0.47**	−0.33
Verbal Memory Delayed	−0.27	**0.45**	−0.27
Info proc 2_1 RT	0.03	−0.22	**0.84**
Info proc 2_2 RT	0.15	−0.16	**0.81**
Info proc 2_3 RT	0.25	−0.34	**0.64**
Info proc 2_3 SD	0.0005	−0.05	**0.48**

Note: NOI1, no interference 1 (letter color); WI, with interference; RT, reaction time; RD, response time difference, WI minus NOI2 (word meaning); SD, standard deviation; Acc, accuracy; NV, non-verbal; rep, repetition; im, immediate; Info proc X_Y, information processing X, number of digits, Y, rate of presentation.

Bold numbers indicate factor loadings corresponding to each specific domain.

### Description of the cognitive tests comprising the cognitive factors

(a) The executive function factor included scores from the Go-NoGo response inhibition test and Stroop interference test. The Go-NoGo test is a continuous performance test of simple RT and response inhibition. In the Stroop interference test performed here, the conflicting information provided by the meaning of a word and the color of its letters leads to a decrement in performance relative to the test phases where there is no conflict. In total, the test includes three phases—phase I (no interference: letter color), phase II (no interference: word meaning), and phase III (interference between letter color and word meaning). The executive function domain is an average of RT in phase III (interference), response time difference (RD) between RT in phase III and phase II, RT in phase I (no interference: letter color) and standard deviations (SD) in phase I (no interference: letter color).
(b) The memory domain included non-verbal and verbal memory tests. The verbal memory test measures immediate and delayed recognition memory for verbal paired associates. In the non-verbal memory test, participants are presented with eight black-and-white drawn pictures of simple common objects or shapes and are instructed to remember their orientation. Each of the tests included four repetitions of the study phase and a recognition test. This is followed by a delayed recognition test after two intervening tests. The memory domain included accuracy on the four immediate repetitions and delayed phase of the non-verbal memory test and accuracy on the first immediate repetition and delayed phase of the verbal memory test.
(c) The staged information processing speed test measures information processing at increasing levels of complexity. The test is comprised of three blocks of information processing load: single digits, two-digit arithmetic problems (e.g., 5 − 1), and three-digit arithmetic problems (e.g., 3 + 2 − 1). For each of these three blocks, stimuli are presented at three different rates (speed levels), incrementally increasing as testing continues. The information processing speed domain is an average of the RT in block 2 (two-digit arithmetic problems) at the three different speed levels and SD of the RT in block 2, level 3.

### MRI acquisition

MR imaging was performed on a 3T (GE) MRI system at the Tel Aviv-Sourasky Medical Center. The MRI protocol included conventional anatomy sequences and DTI acquired with a standard head-coil.

#### Conventional anatomy sequences

T_1_-weighted images: 3D spoiled gradient recalled echo (SPGR) sequence with the following parameters: 66 axial slices, TR/TE = 400/3.2 ms, and resolution of 1 × 1 × 2 mm^3^, scan time of 4 min.

#### DTI protocol

Spin-echo diffusion weighted echo planar imaging (DW-EPI) sequence was performed with 48 axial slices and resolution of 2.5 × 2.5 × 2.5 mm^3^. Diffusion parameters were: TR/TE = 25/19 ms, *b*-value of 1000 s/mm^2^ acquired with 19 gradient directions. The DW-EPI sequence was gated to the cardiac cycle with TR of 30 R–R intervals and TE of 88 ms.

### Image analysis

Correction of head motion image artifacts, normalization, and statistical analysis were performed using the SPM software (version 2, UCL, London, UK). The image analysis routine of the diffusion tensor data included the following steps:

1. All of the DWI images were coregistered using SPM2 (UCL, London, UK) to correct for head motion. Gradient orientations were compensated prior to the b-matrices calculation to account for the rotation component of the registration.
2. Spatial normalization was applied: images were normalized to the MNI coordinates, using non-linear deformations in SPM2, and compensating the gradients orientations for the rotation component of the affine-transformation that is the closest to the non-linear deformations. Normalization was performed using a 12-parameter affine transformation followed by non-linear transformations, with 1176 parameters describing each deformation field.

### Fiber tracking procedure

Tensor fitting was performed using the free water elimination method (Pasternak et al., 2009). The free water elimination method allows correcting for water contamination, especially in brain tissue adjacent to the lateral ventricles, for example the fornix. It is achieved by fitting a bi-tensor model for which a mathematical framework is introduced to stabilize the fitting. In this routine, we eliminate a tensor component that fits the diffusion properties of free water and use for tracking only the fiber component of the tensor. The free water elimination is important when focusing on elderly subjects as in the present work, since atrophy and enlarged ventricles might result in lower FA values in white matter structures such as the fornix, which is adjacent to the lateral ventricles.

The tensors obtained were spectrally decomposed to their eigen-components. The eigenvalues were used to calculate FA maps (Basser and Pierpaoli, 1998). Tractography was applied using the principal eigenvectors and FA: the brute force FACT algorithm was used to generate the fiber coordinates, terminating at voxels with FA lower than 0.2 or following tract orientation change higher than 60°. Fibers that passed through a manually chosen seed region of interest (ROI) were plotted. The fibers were plotted as streamlines using Matlab (Mathworks, Natick, MA). Once a subset of fibers had been found, a visitation map was generated, indicating voxels with at least one streamline passing through. The masks obtained were overlaid over the B_0_ image. Overall 10 fibers tracts were plotted for each subject: five temporal projections (UF, fornix, cingulum, SLF, ILF), in both hemispheres.

**Figure 1:**
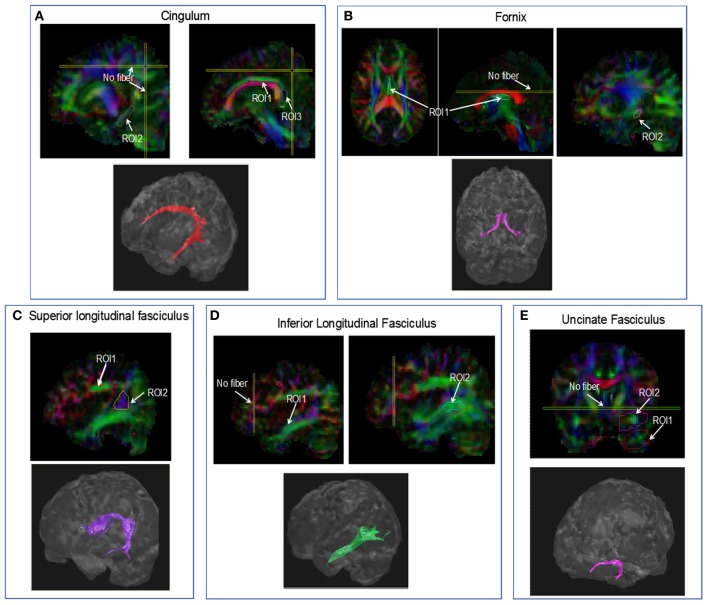
**Fiber tracking procedure.** Representative seed ROIs drawn on the DTI color coded map, below the plotted fiber bundles for each temporal projection. **(A)** cingulum, **(B)** fornix, **(C)** SLF, **(D)** ILF, and **(E)** uncinate fasciculus.

Representative seed ROIs are shown in Figure 1, as well as the reconstructed fiber. The seed ROIs were chosen based on visual comparison with the MRI atlas of human white matter (Mori and Crain, 2005). The seed ROIs were drawn on the DTI color map for each temporal projection in the following manner:

1. The Cingulum: Three ROIs were used in order to reconstruct the cingulum bundle. Two seed ROIs were drawn in the mid-sagittal plane: first ROI within the body of cingulum (Figure 1A, ROI 1), second ROI in the posterior part of the cingulum (Figure 1A, ROI3). Another was drawn in a more lateral sagittal slice in the temporal part of the cingulum adjacent to the hippocampus (Figure 1A, ROI2). If necessary, in order to eliminate fibers which are not part of the cingulum, a “no-fiber” plane was drawn in a coronal plane posterior to the cingulum and in an axial plane dorsal to the cingulum (Figure 1A, no fiber).
2. The Fornix: two/three ROIs were used to reconstruct the fornix. First ROI was drawn on an axial plane, marking the body of the fornix (Figure 1B, ROI1). Second ROI was drawn on the temporal part of the fornix (Figure 1B, ROI2). If necessary, a third ROI was drawn in the axial plane anterior to the splenium of the CC. In order to eliminate fibers which are not part of the fornix, a “no-fiber” plane was drawn in an axial plane dorsal to the fornix (Figure 1B, no fiber).
3. The SLF: two ROIs were used to reconstruct the SLF. First ROI was drawn in a sagittal plane in the fronto-parietal part of the SLF (Figure 1C, ROI1) and a second ROI was drawn in the parieto-temporal part of the SLF (Figure 1C, ROI2).
4. The ILF: two ROIs were used to reconstruct the ILF in a sagittal plane. First ROI was drawn in the occipital (posterior) part of the ILF (Figure 1D, ROI1). Second ROI was drawn in the temporal (anterior) part of the ILF (Figure 1D, ROI2). If necessary, in order to eliminate fibers which are not part of the ILF, a “no-fiber” plane was drawn in a coronal plane anterior to the ILF (Figure 1D, no fiber).
5. The UF: two ROIs were used to reconstruct the UF in a coronal plane. First ROI was drawn in the temporal part of the UF (Figure 1E, ROI1), and a second ROI was drawn in the frontal part of the UF (Figure 1E, ROI2). If necessary, in order to eliminate fibers which are not part of the UF, a “no-fiber” plane was drawn in an axial slice dorsal to the frontal part of the ILF (Figure 1E, no-fiber).

### Creation of tract ROIs and registration of DTI maps

1. After the tracking procedure, a mask was created from the tracts matrices of all subjects in order to create a tract mask. Overall 10 masks were created for each subject: five temporal projections (UF, fornix, cingulum, SLF, ILF), in both hemispheres, right and left. For each temporal projection, the tract masks (that are already normalized to the MNI template) of the different subjects were registered to a tract mask of one young subject. The registration procedure was performed using normalized mutual information (Studholme et al., 1999) in the SPM software (version 2, UCL, London, UK). Spatial registration involved finding parameters that either maximize or minimize the normalized mutual information function, followed by a voxel to voxel affine transformation.
2. The registered tract masks of all subjects were summed to create the total tract mask. For each tract only voxels that 80% of the subjects had a tract passing through them were included to create the final fiber ROI.
3. The same registration parameters that were created for each temporal projection were applied (right and left) on the DTI maps (ADC, FA).
4. In order to apply the random field theory (RFT) Spatial smoothing with 8 mm full width half maximum Gaussian kernel was performed.

### Statistical analysis

Voxel-based analysis: VBA is a whole brain technique that allows the detection of regionally specific differences in brain tissue composition on a voxel-by-voxel basis (Ashburner and Friston, 2000). In this work we used SPM (version 2, UCL, London, UK) to perform voxel-by-voxel correlation between the DTI indices and performance in each of the cognitive domains. For each temporal projection, simple regression was performed between the DTI indices (ADC, FA, D_A_, D_R_) and age in each temporal projection and multiple regressions were performed between the DTI indices (ADC, FA, D_A_, D_R_) co-registered to the appropriate temporal projection, and scores in the four cognitive domains (executive function, information processing speed, and memory). The multiple regressions were controlled for age and motor ability using the multiple regression routine in SPM with age and scores in a simple finer tapping task were used as constancies. The fiber ROI was used as a mask. The statistical threshold was set at *p* < 0.05, after Hochberg sequential correction for multiple comparisons (Hochberg, 1988).

The multiple regression analysis generated statistical parametric maps. The multiple regression routine included applying implicit and explicit masks (the appropriate fiber ROI, see above), without applying grand mean scaling, threshold masking, or global calculation. Correction for multiple comparisons was performed on the statistical parametric map, and the five ROIs were used to constrain the analysis—reducing the multiple comparison correction to number of voxels contained within the 10 ROIs (5 tracts on L and R sides). The statistical parametric maps are presented superimposed on an average T1 image of all subjects, with the fiber ROIs drawn upon them allowing an anatomical informative reference.

Scatter plots of scores of the cognitive domains and the averaged DTI parameters extracted only in the voxels passing the significance threshold are presented. A linear fit (*y* = *mx* + *b*) was performed and the best linear fit is shown on the scatterplots.

## Results

Factor analysis of the cognitive tests resulted in three cognitive domains: the memory domain, the executive function domain, and the information processing speed domain. Significant correlations between age and cognitive performance were obtained, as reported previously (Sasson et al., 2012).

**Figure 2:**
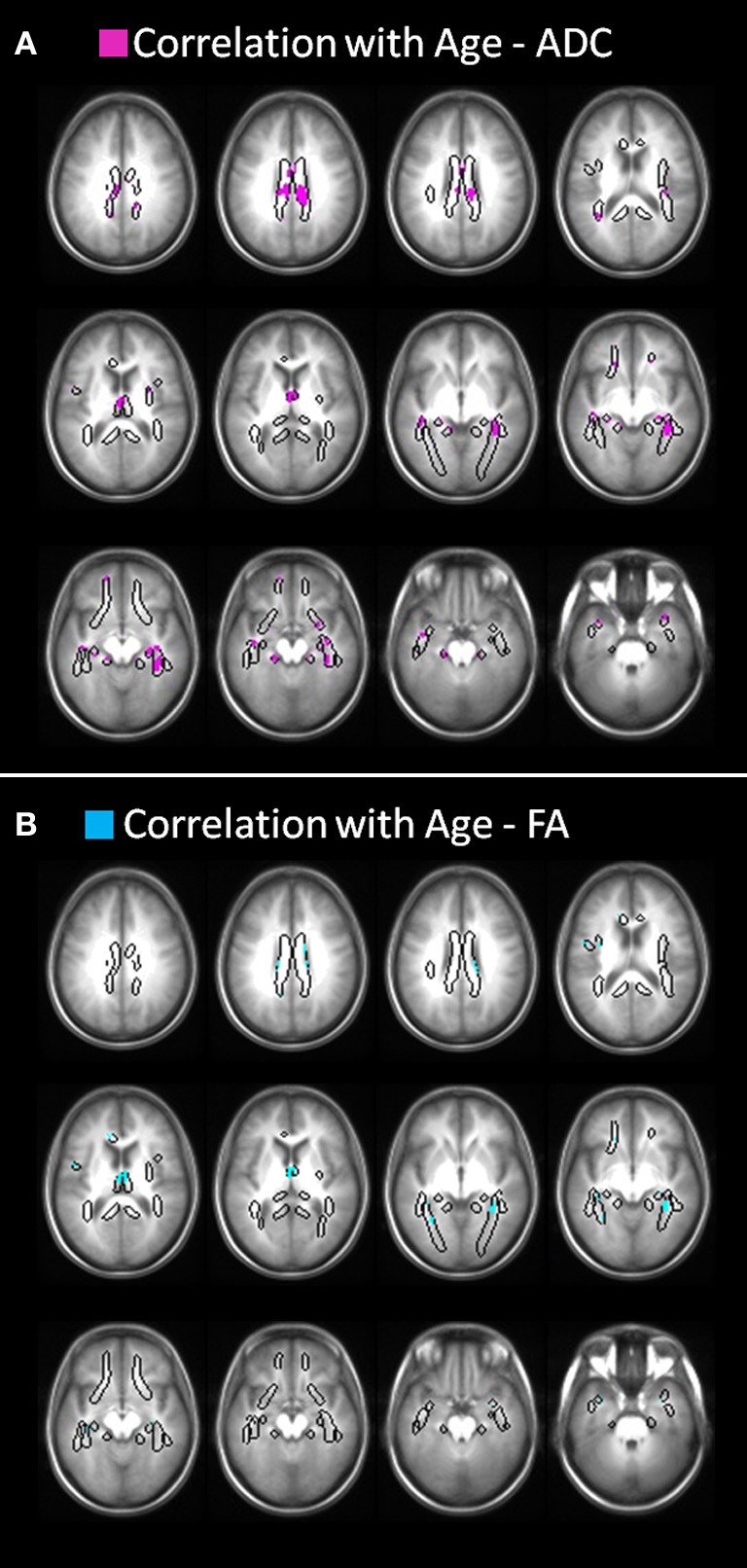
**DTI correlates of age.(A)** Positive correlation between age and ADC (*p* < 0.05, corrected). Correlations were found in all fibers, including bilateral cingulum, SLF, fornix, ILF, and UF. Correlation is found in frontal, temporal, and parietal parts of the fibers. **(B)** Negative correlation between age and FA (*p* < 0.05, uncorrected). FA showed less significant voxels correlated with age compared to ADC, however, correlation was found in all fibers accept right SLF, which did not pass the correction for multiple comparisons.

Positive regression was obtained between age and ADC (*p* < 0.05, corrected) in all fibers, including bilateral cingulum, SLF, fornix, ILF, and UF. Significant positive regression with age is found mostly in frontal, temporal, and parietal parts of the fibers and not in occipital parts [Figure 2A Negative regression between age and FA (*p* < 0.05, corrected) was found in all fibers accept right SLF, which did not pass the correction for multiple comparisons (Figure 2B)]. Regression between FA and age was more prominent in the fornix and in temporal part of the ILF and less in the cingulum, SLF and UF.

**Figure 3:**
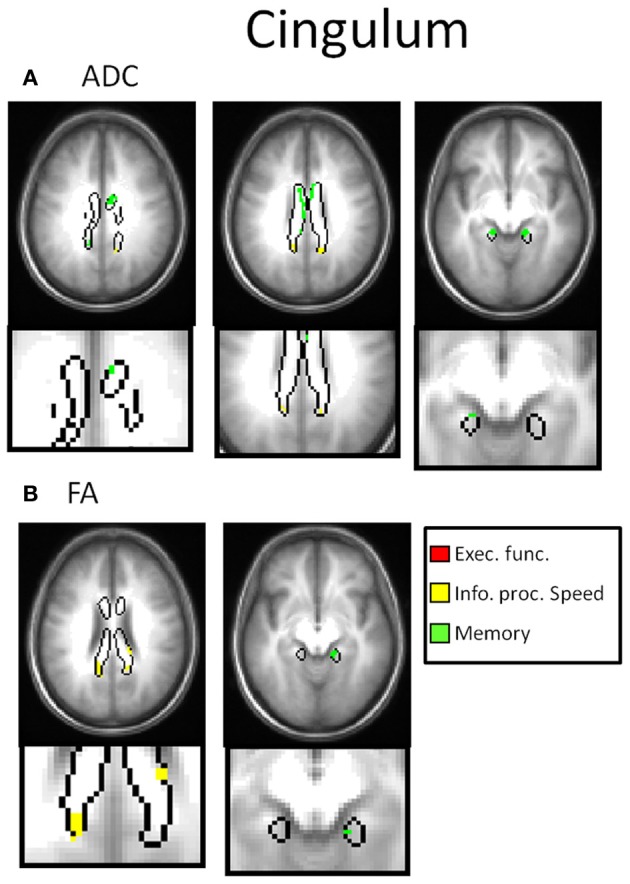
**DTI correlates of cognitive performance in the cingulum.** In the upper row, parametric maps of the multiple regression analysis between performance in the cognitive domains and DTI parameters (ADC and FA) corrected for age and motor ability (*p* < 0.05, uncorrected) and in the lower row, magnification of the significant clusters that passed the correction for multiple comparisons (*p* < 0.05, corrected). **(A)** Correlation with ADC. In the right cingulum, negative correlation was obtained between performance in the memory domain with ADC (green clusters). **(B)** Correlation with FA. In the left cingulum correlation was obtained between performance in the information processing speed domain and FA (yellow clusters). No correlation was obtained between ADC nor FA with executive function (red clusters).

**Figure 4:**
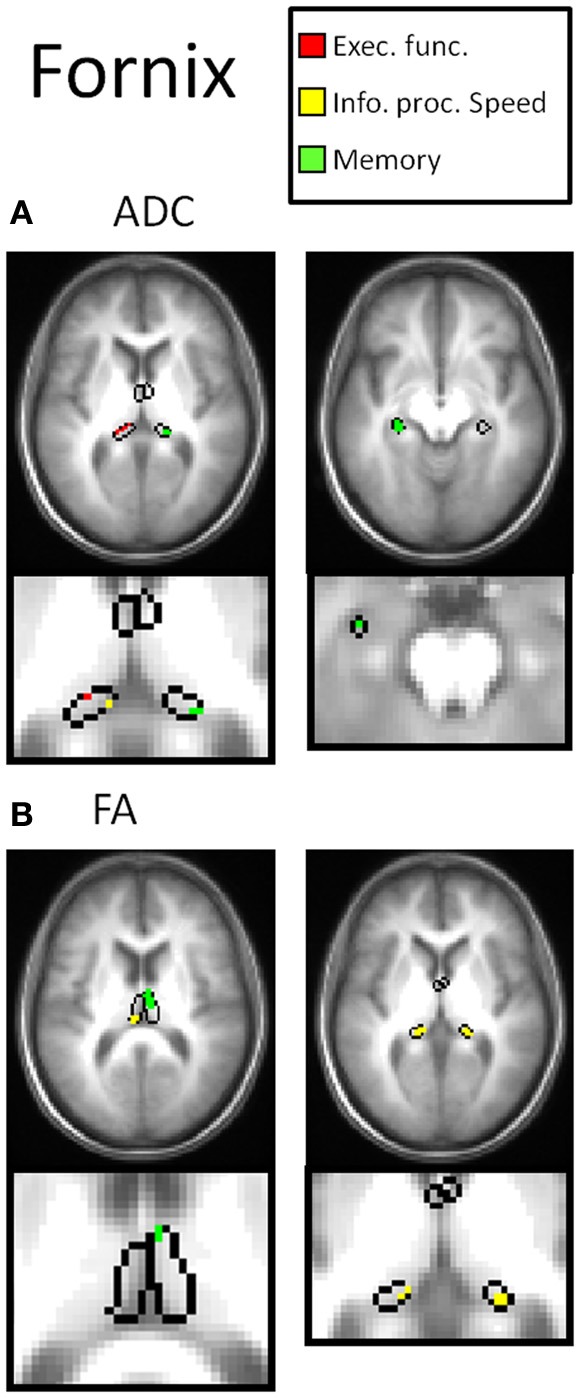
**DTI correlates of cognitive performance in the fornix.** In the upper row, parametric maps of the multiple regression analysis between performance in the cognitive domains and DTI parameters (ADC and FA) corrected for age and motor ability (*p* < 0.05, uncorrected) and in the lower row, magnification of the significant clusters that passed the correction for multiple comparisons (*p* < 0.05, corrected). **(A)** In the right fornix, negative correlation was obtained between performance in the memory domain with ADC (green clusters). Positive correlation was obtained in left fornix between executive function and ADC, however the clusters did not pass the correction for multiple comparisons (red clusters). **(B)** In bilateral fornix between performance in the information processing speed domains with FA (yellow clusters).

**Figure 5:**
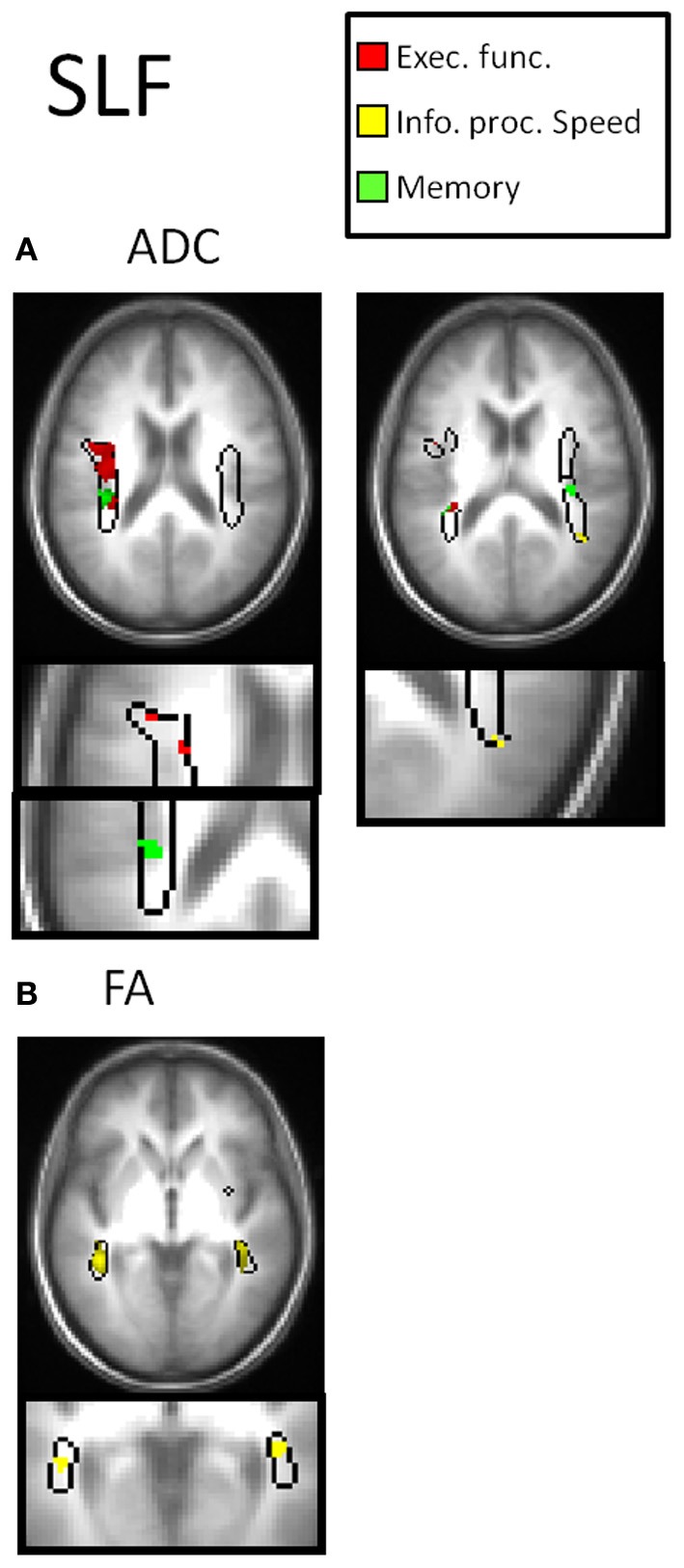
**DTI correlates of cognitive performance in the SLF.** In the upper row, parametric maps of the multiple regression analysis between performance in the cognitive domains and DTI parameters (ADC and FA) corrected for age and motor ability (*p* < 0.05, uncorrected) and in the lower row, magnification of the significant clusters that passed the correction for multiple comparisons (*p* < 0.05, corrected). **(A)** Correlation with ADC. In the left SLF, negative correlation was obtained between performance in the executive function domain with ADC (red clusters) and positive correlation between memory domain with ADC (green clusters). **(B)** Correlation with FA. In the right SLF negative correlation was obtained between performance in the information processing speed domain and FA (yellow clusters).

**Figure 6:**
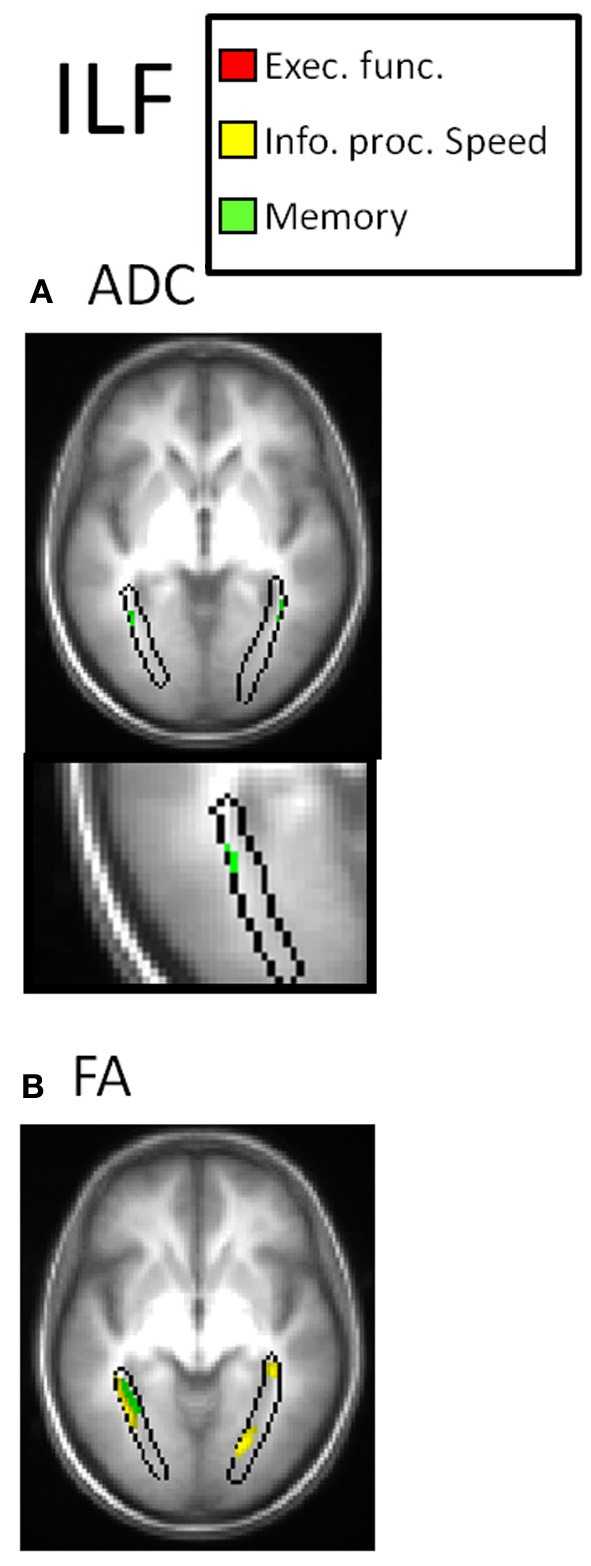
**DTI correlates of cognitive performance in the ILF.** In the upper row, parametric maps of the multiple regression analysis between performance in the cognitive domains and DTI parameters (ADC and FA) corrected for age and motor ability (*p* < 0.05, uncorrected) and in the lower row, magnification of the significant clusters that passed the correction for multiple comparisons (*p* < 0.05, corrected). **(A)** Correlation with ADC. In the left ILF, positive correlation was obtained between performance in the memory domain and FA (green clusters). **(B)** Correlation with FA. In bilateral ILF negative correlation was obtained between performance in the information processing speed domain and FA (yellow clusters). No correlation was obtained between ADC nor FA with executive function (red clusters).

**Figure 7:**
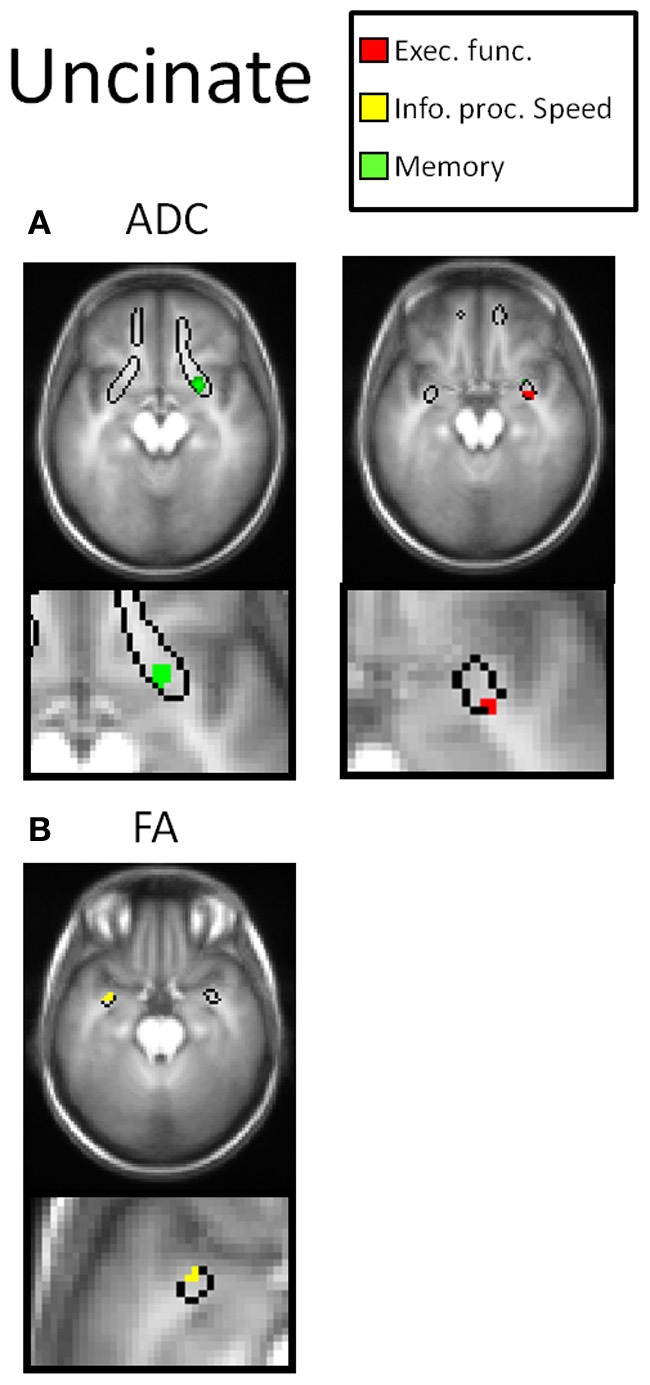
**DTI correlates of cognitive performance in the UF.** In the upper row, parametric maps of the multiple regression analysis between performance in the cognitive domains and DTI parameters (ADC and FA) corrected for age and motor ability (*p* < 0.05, uncorrected) and in the lower row, magnification of the significant clusters that passed the correction for multiple comparisons (*p* < 0.05, corrected). **(A)** Correlation with ADC. In the right UF negative correlation was obtained between performance in the memory domain with ADC (green clusters) and positive correlation in the executive function domains with ADC (red clusters). **(B)** Correlation with FA. In the left UF positive correlation was obtained between performance in the information processing speed domain and FA (yellow clusters).

Table 2 and Figures 3–7 summarize the results of the multiple regression analysis between performance in the cognitive domains and DTI parameters corrected for age and motor ability. In Figures 3–7, the statistical parametric maps of the partial correlation between ADC/FA and scores in the cognitive domains in each temporal projection corrected for age and motor ability are shown (*p* < 0.05, uncorrected) and below magnification of the significant clusters that passed the correction for multiple comparisons (*p* < 0.05, corrected). In Table 2, only significant voxels that passed the statistical threshold (*p* < 0.05, corrected) are presented. Partial correlation between axial and radial diffusivities and scores in the cognitive domains are also reported below.

**Table 2:** **Partial correlation between DTI parameters: ADC, FA, axial diffusivity (D_A_) and radial diffusivity (D_R_) and performance in the cognitive domains corrected for age and motor ability (*p* < 0.05, corrected)**.

			**Executive function**	**Information processing speed**	**Memory**
Fornix	ADC	R			+
		L			
	FA	R			
		L		+	
	D_A_	R			
		L			
	D_R_	R			+
		L			
Cingulum	ADC	R			
		L			+
	FA	R			
		L		+	
	D_A_	R			
		L			
	D_R_	R			
		L			
ILF	ADC	R			
		L			
	FA	R		+	
		L		+	+
	D_R_	R			
		L			+
	D_A_	R			
		L			
SLF	ADC	R			
		L	+		+
	FA	R		+	
		L		+	
	D_R_	R			+
		L			+
	D_A_	R			
		L	+		
Uncinate	ADC	R	+		+
		L			
	FA	R			
		L			
	D_R_	R			
		L			
	D_A_	R	+		
		L			

ILF, inferior longitudinal fasciculus; SLF, superior longitudinal fasciculus; R, right; L, left.

**Figure 8:**
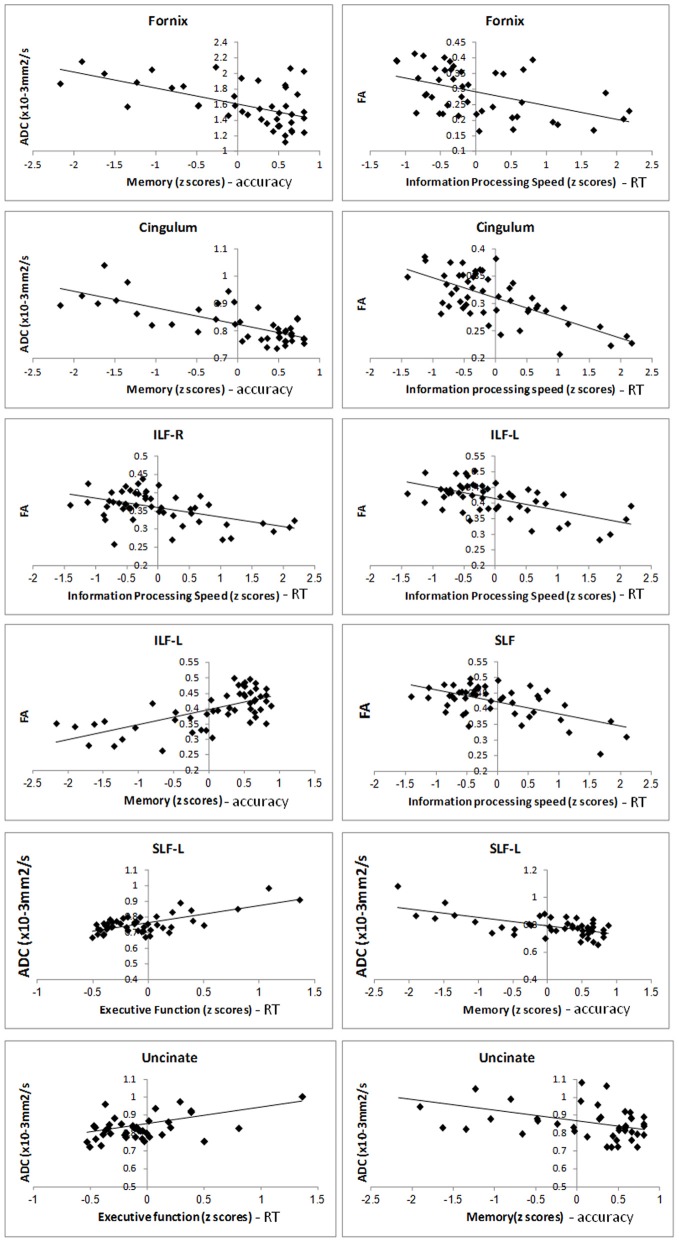
**Scatter plots of the DTI parameters and scores in the cognitive domains.** Scatter plots of DTI parameters extracted from the significant clusters (in Y-axis) and *Z*-scores in the cognitive domains (in the X-axis).

In Figure 8, scatter plots of scores of the cognitive domains and the averaged DTI parameters extracted only in the voxels passing the significance threshold are presented. A linear fit (*y* = *mx* + *b*) was performed and the best linear fit is shown on the scatterplots. In the right cingulum, negative partial correlation was found between performance in the memory domain (accuracy scores) with ADC in frontal as well as temporal parts of the cingulum (Figure 3A) and with D_A_. In the left cingulum negative partial correlation was obtained between performance in the information processing speed domain (RT scores) and FA in parietal part of the cingulum (Figure 3B).

In the right fornix, negative correlation was obtained between performance in the memory domain (accuracy scores) with ADC (Figure 4A) and with radial diffusivity (D_R_), and in bilateral fornix between performance in the information processing speed (RT scores) domains with FA (Figure 4B). Correlations were found mainly in temporal parts of the fornix.

In the left SLF, negative partial correlation was obtained between performance in the executive function domain (RT scores) with ADC, D_A_ and D_R_ and in frontal parts of the SLF positive correlation between memory domain (accuracy scores) with ADC (Figure 5A) and D_R_; in the right SLF negative correlation was obtained between performance in the information processing speed domain (RT scores) and FA in parietal part of the SLF (Figure 5B).

In the left ILF, positive correlation was obtained between performance in the memory domain (accuracy scores) and FA and positive correlation with D_R_ in temporal parts of the ILF (Figure 6B). In bilateral ILF negative correlation was obtained between performance in the information processing speed domain (RT scores) and FA in occipital parts of the ILF (Figure 6B).

In the right UF negative correlation was obtained between performance in the memory domain (accuracy scores) with ADC and positive correlation in the executive function domain (RT scores) with ADC in temporal parts of the UF (Figure 7A).

In the left UF positive correlation was obtained between performance in the information processing speed domain (RT scores) with FA in temporal parts of the UF (Figure 7B).

## Discussion and conclusions

The main finding of the present study is that WM integrity is correlated with cognitive performance in a fiber specific manner.

In conclusion the results revealed that performance in the executive function domain (RT) is correlated positively with ADC and negatively with FA in the left SLF and right UF; performance in the information processing speed domain (RT) is correlated with negatively with FA in the left cingulum, left fornix, right and left ILF and SLF; and the memory domain (accuracy) shows significant positive correlations with FA and negative correlations with ADC in the right fornix, right cingulum, left ILF, left SLF, and right UF.

DTI tractography was performed using the free water elimination method in order to minimize the partial volume. In the correlation analysis we used partial correlation correcting for age and motor ability, in order to isolate correlations more specific to each of the cognitive domains.

Functionality has been heavily studied in gray matter regions. However, functionality in white matter fiber bundles has only recently been investigated. Indeed only in the last 10 years DTI tractography enabled us to investigate functionality of white matter fiber bundles. Our approach, using factor analysis of a battery of cognitive tests, allows us to localize functional domains in distinctive white matter fiber tracts.

Most of our findings are consistent with previous studies and a summary of our findings in each temporal projection, as well as the known literature on the functionality of the distinctive white matter tracts and surrounding regions, as summarized in Table 3.

**Table 3:** **A summary of cognitive localization in the temporal projections obtained in the present study along with the known literature**.

	**Cognitive domains**	**Previous findings**	**References**
Cingulum	Memory	Working memory, attention	Valenstein et al., 1987; Charlton et al., 2006; Sepulcre et al., 2009
Fornix	Memory	Memory	Spiers et al., 2001
SLF	Executive function (L, frontal) Information processing speed (R, parietal)	Higher brain functions, Language (Uncinate) Calculation (parietal lobe)	Lichtheim, 1885; Geschwind, 1965a,b; Heilman et al., 1970; Tanabe et al., 1987; Rocha et al., 2005; Cavanna and Trimble, 2006
ILF	Memory	Visual memory	Bauer and Trobe, 1984; Shinoura et al., 2007; Thomas et al., 2009; Tavor et al., 2010 Antonova et al., 2005; Hu et al., 2011
UF	Information processing speed Memory	Memory	Nakamura et al., 2005; Di Paola et al., 2011; O'Dwyer et al., 2011

### Cognitive performance localization in the cingulum

A significant negative correlation was obtained in the right anterior cingulum between ADC and performance in the memory domain. This finding is consistent with previous reports of involvement of the anterior cingulate and cingulum in memory performance (Valenstein et al., 1987; Sepulcre et al., 2009; Charlton et al., 2010). A negative correlation was also obtained between ADC in the posterior cingulum with performance in the memory domain.

### Cognitive performance localization in the fornix

Lesion studies suggest that the fornix, which includes cholinergic afferents to the hippocampus, is important for learning and formation of new memories (Spiers et al., 2001).

Indeed in the present study we obtained that ADC in the right fornix is significantly negatively correlated with performance in the memory domain.

### Cognitive performance localization in the superior longitudinal fasciculus

Various studies suggest an important role for the SLF in higher brain functions, and particularly language (Lichtheim, 1885; Damasio and Damasio, 1980; Tanabe et al., 1987; Catani et al., 2003; Wise, 2003; Geldmacher et al., 2007). We obtained a negative correlation between performances in the information processing speed domain, with bilateral FA in the SLF. The correlations were localized to parietal regions within the right SLF. The parietal lobe is well known from the literature to be related to calculation (Rocha et al., 2005; Cavanna and Trimble, 2006).

A strong significant positive correlation was also obtained between performance in the executive function domain and ADC in the left SLF. The Arcuate fasciculus (AF), a part of the SLF, has been central to the neurobiological interpretation of higher brain function generally and for language in particular (Lichtheim, 1885; Geschwind, 1965a,b; Heilman et al., 1970; Tanabe et al., 1987). The task we used here, stroop interference involves interference between semantic information and visual input, and hence involves linguistic abilities, and is also considered as a higher function task.

### Cognitive performance localization in the inferior longitudinal fasciculus

The ILF is known to play an important role in visual memory as demonstrated by post mortem studies (Bauer and Trobe, 1984) lesion studies (Shinoura et al., 2007) and imaging studies. In congenital prosopagnosia correlation was found between face recognition and FA in the ILF (Thomas et al., 2009; Tavor et al., 2010). In our work we found a significant positive correlation between memory performance and FA in the left ILF. The memory tasks were visual tasks and included visual spatial memory and visual verbal memory.

There was also negative correlation between FA in the left ILF, and performance in the information processing speed domain. There are some findings in the literature supporting an association between measurements of occipital white matter structures with information processing speed. Smaller occipital white matter volume was associated with slower information processing speed in schizophrenic patients (Antonova et al., 2005). Higher average fractional anisotropy (FA) in left occipitotemporal junction was obtained in children trained in mental calculation (Hu et al., 2011).

### Cognitive performance localization in the uncinate fasciculus

The UF plays an important role in the formation and retrieval of memories as shown by lesion (Levine et al., 1998) and animal studies (Squire et al., 2004). Indeed, we found a negative correlation between ADC in the right UF with performance in the memory domain. In a study using diffusion tensor imaging (DTI) (using tract based statistics—TBSS) increased axial diffusivity was found in the UF in patients with amnestic mild cognitive impairment (O'Dwyer et al., 2011). Lower white matter density was found in the UF (as well as in the fornix and anterior cingulum) in patients with hypoxic amnesia (Di Paola et al., 2011). Lesion studies have demonstrated that the frontal lobe is crucial for executive functions (Milner, 1971; Drewe, 1974; Perret, 1974; Rowe et al., 2001). Indeed, the ADC values in the UF which projects into the pre-frontal cortex were obtained in the present study to correlate with performance in the executive function domain. This finding is consistent with previous imaging studies; FA in the UF was correlated with executive function in subjects with schizotypal personality disorder (Nakamura et al., 2005).

Lower ADC and higher FA imply on higher white matter integrity, and indeed in the present paper, they were correlated with higher accuracy and lower RT. Higher FA values may indicate higher tissue directionality and organization, increased axonal and myelin density. Lower ADC values may indicate higher tissue density (Gouw et al., 2008; Blumenfeld-Katzir et al., 2011). Postmortem studies have shown that aging is accompanied by loss of 25–45% of the total length of myelinated fibers in the brain white matter (Marner et al., 2003). Degeneration of myelin sheaths and loss of nerve fibers in several but not all fiber bundles were correlated with cognitive decline in monkeys, among them the fornix (Peters and Kemper, 2011). Therefore, it is hard to interpret to which biological mechanism to relate the correlation of DTI parameters with cognitive decline. Based on the literature, it can be attributed to axonal loss, degeneration of myelin sheaths, loss of oligodendrocytes (Bartzokis, 2004), or other glial cells.

Correlation with ADC was accompanied by correlation with either D_R_, D_A_ or both; however, correlation with FA did not correspond with correlation with either diffusivity except in the right ILF in which correlation between memory performance and D_R_ was obtained. This can be explained by the fact that ADC is an average of both diffusivities; however, change in FA is a result of change in the variance between the diffusivities.

## Limitations of the present study

DTI used in the present study has several methodological limitations. Reduced FA and an increase in ADC can result from partial volume with the surrounding tissue. In the present study we used the free water elimination method (Pasternak et al., 2009) to address this issue. The tracking procedure itself has several limitations. First, crossing fibers cause a reduction in FA and erroneous termination of a tract. Second, the fiber tracking procedure is subjective, and ROI placement can differ among observers. Further, imperfect registration of the fiber tracts can be problematic when performing the analysis using group voxel-based statistics and may include voxels that are not part of the fiber tract. Partial volume with surrounding tissue may also result from the smoothing procedure.

## Summary

In conclusion, using DTI tractography, cognitive performance can be correlated with WM integrity in the temporal projections. Combining voxel wise correlation analysis and fiber tracking, enables anatomical definition of ROI for correlation analysis of behavioral parameters with diffusion indices, and functionality can be correlated with white matter integrity.

### Conflict of interest statement

The authors declare that the research was conducted in the absence of any commercial or financial relationships that could be construed as a potential conflict of interest.
